# Automatic Analysis of Composite Physical Signals Using Non-Negative Factorization and Information Criterion

**DOI:** 10.1371/journal.pone.0032352

**Published:** 2012-03-01

**Authors:** Kenji Watanabe, Akinori Hidaka, Nobuyuki Otsu, Takio Kurita

**Affiliations:** 1 National Institute of Advanced Industrial Science and Technology (AIST), Tsukuba, Ibaraki, Japan; 2 School of Science and Engineering, Tokyo Denki University, Hiki, Saitama, Japan; 3 Faculty of Engineering, Hiroshima University, Higashi-Hiroshima, Hiroshima, Japan; Wayne State University, United States of America

## Abstract

In time-resolved spectroscopy, composite signal sequences representing energy transfer in fluorescence materials are measured, and the physical characteristics of the materials are analyzed. Each signal sequence is represented by a sum of non-negative signal components, which are expressed by model functions. For analyzing the physical characteristics of a measured signal sequence, the parameters of the model functions are estimated. Furthermore, in order to quantitatively analyze real measurement data and to reduce the risk of improper decisions, it is necessary to obtain the statistical characteristics from several sequences rather than just a single sequence. In the present paper, we propose an automatic method by which to analyze composite signals using non-negative factorization and an information criterion. The proposed method decomposes the composite signal sequences using non-negative factorization subjected to parametric base functions. The number of components (i.e., rank) is also estimated using Akaike's information criterion. Experiments using simulated and real data reveal that the proposed method automatically estimates the acceptable ranks and parameters.

## Introduction

Time-resolved spectroscopy is widely used for analysis in fields such as chemistry and biology [1]–[4]. In this form of spectroscopy, energy transfer from an excited state of fluorescence materials [1], [2] or free diffusion of materials [3], [4] in thermal equilibrium is measured as a signal sequence in order to analyze the physical characteristics of the materials. The signal sequence (usually, a composite physical signal) is represented as a sum of non-negative signal components [1]–[4]. The signal components represent kinetic energy distributions for each energy level, and the physical characteristics of the materials are estimated from the parameters of the components (such as existence ratios and energy levels), which are usually calculated using fitting methods [1]–[4]. In a signal sequence for free diffusion in particular, the energy levels for each component are related to the diffusion times of materials [3], [4]. Thus, in order to analyze the physical characteristics of the materials, the parameters of model functions that represent the energy dynamics in thermal equilibrium (i.e., the Boltzmann distribution) are usually estimated from each measured signal sequence [1]–[4]. In this case, it is often necessary to obtain the statistical characteristics from several sequences rather than just a single sequence [1]–[4] in order to quantitatively analyze real measurement data. In many such analyses, the number of components was manually decided so as to be explainable according to domain-specific knowledge (heuristics) obtained from chemical, biological, and/or physical experiments [3], [4]. In order to quantitatively analyze real data, it is desirable that the number of components is automatically decided in order to reduce the risks of subjective decisions, because the estimated physical parameters of the components change depending on the number of components. As such, it is appropriate to apply statistical methods to multiple signal sequences. Such a statistical analysis method for spectroscopic measurement data would contribute to improved analysis accuracy in a wide range of chemical and biological research fields [1]–[4].

Signal factorization methods, such as factor analysis, principal component analysis (PCA) [5], independent component analysis (ICA) [6], [7], and positive or non-negative matrix factorization (PMF [8] or NMF [9], [10]), have been applied to a range of fields. In particular, NMF used together with a fitting method [11] is effective for factorizing non-negative energy distributions, such as the Boltzmann distribution, because the energy distribution can be represented as a positively weighted sum of a few non-negative components. These components are not necessarily orthogonal. On the other hand, PCA and ICA are not suitable for this purpose because they do not exhibit non-negativity. Actually, PCA decomposes the signals into a sum of orthogonal basis vectors. Non-negativity was introduced to ICA by Plumbley [12], and non-negative ICA will be effective for the estimation of source signals based on observed signals. However, non-negative ICA does not consider the non-negative constraint for the mixing matrix [12]. When the mixing matrix includes negative values, the measured signal is represented as a sum of negative and non-negative source signal components, despite the fact that the composite physical signal consists of a non-negative sum of non-negative components.

Boltzmann non-negative matrix factorization (BzNMF) [13] is an effective method for analyzing composite physical signals that are subject to the Boltzmann distribution law. BzNMF decomposes an input matrix (i.e., a set of non-negative signal sequence vectors) into non-negative basis vectors under the constraint that the decomposed basis vectors are represented by the Boltzmann distribution. BzNMF can be used to estimate statistical and physical parameters from a set of input signal sequences. Physical parameter estimation by BzNMF is more applicable to a wider range of energy kinetics analysis than fitting methods, such as using the Fourier transform of a time-series concentration transition. This is because the Boltzmann distribution often represents the basic energy kinetics distribution in chemistry and physics. In [13], the objective function of BzNMF was defined by the generalized Kullback-Leibler (KL) divergence. However, in physical chemistry [14], optimization problems are usually solved using the method of least square error (LSE).

In the present paper, we propose an automatic analysis method for composite physical signals, which are represented as the sum of the Boltzmann distributions. The proposed method decomposes the composite signals using BzNMF, which is optimized in LSE. The number of components (i.e., the rank) is also estimated using Akaike's information criterion (AIC) [15] in order to reduce the risk of improper rank estimation.

We confirmed the validity of the proposed method by conducting experiments using simulation data and real data. The simulation data were generated using the sum of the Boltzmann distributions, and the real data are for standard samples measured by fluorescence correlation spectroscopy (FCS) [16]–[18] in [3]: Chemical particle (rhodamine 6G: Rh6G) and fluorescence protein (enhanced green fluorescence protein: EGFP).

## Methods

### Composite physical signals

In time-resolved spectroscopy, when we measure energy transfer from an excited state of fluorescence materials [1], [2] or free diffusion of materials [3], [4] in thermal equilibrium, the *i*-th signal intensity (1≤*i*≤*N*) at the *j*-th measurement time point (1≤*j*≤*M*) *I* ^(*i*)^(*t_j_*) = *I_j_* obtained by approximating the sum of exponential functions is defined as follows:
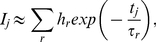
(1)where *h_r_* denotes a non-negative coefficient of the *r*-th system (component). *τ_r_* (>0) is the *r*-th time constant, such as the energy transfer time or the diffusion time of the materials, and *t_j_* (>0) is the *j*-th measurement time point.

In physics, the energy transfer in thermal equilibrium is expressed by the Boltzmann distribution law. The sum of the Boltzmann distributions *p*(*ε_j_*) is not usually expressed as in Eq. (1), but is instead defined as
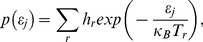
(2)where the quantity *ε_j_* represents the difference between the *j*-th energy level and the lowest energy level. The three parameters *h_r_*, *κ_B_*, and *T_r_* are the *r*-th amplitude, the Boltzmann constant, and the absolute temperature of the *r*-th component, respectively.

When we measure the changes in energy using spectroscopic methods, the quantity *ε_j_* is proportional to the number of measured photons at the *j*-th time point, and the number of photons per unit time is constant. Therefore, *ε_j_* is proportional to *t_j_*. The denominator *κ_B_T_r_* denotes the quantity of heat in the *r*-th component. Therefore, *κ_B_T_r_* must be proportional to the kinetic energy *E_r_* in the *r*-th component, and *E_r_* can then be represented as
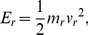
(3)where *m_r_* is the mass of the measurement target of the *r*-th component, and *v_r_* is the velocity of the measurement target of the *r*-th component. When the measurement volume is sufficiently small, *v_r_* can be regarded as being approximately constant, and *E_r_* is proportional to *m_r_*. The *r*-th time constant *τ_r_* for the energy dynamics is also proportional to *m_r_*. Therefore, *κ_B_T_r_* is proportional to *τ_r_*.

Based on the above considerations, the measured signal *I_j_* is expressed as a non-negative linear combination of non-negative components, which are represented by the Boltzmann distribution shown in Eq. (1).

### Non-negative Factorization

In the factorization of composite physical signals, the *M* × *N* input matrix ***I*** is constructed from *N* signal sequences that are measured at *M* time points. The input matrix ***I*** should be decomposed into the given *R*-rank inner products of non-negative basis vectors and non-negative coefficients, because the measured signal sequence is expected to be a non-negative linear combination of certain signal components. NMF [9], [10] was proposed as a means of decomposing a given input matrix ***I*** into an *M* × *R* basis matrix ***W*** = [***w***_1_, …, ***w*** *_R_*] and an *R* × *N* coefficient matrix ***H*** = [***h***_1_, …, ***h*** *_N_*], as follows:
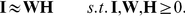
(4)This means that ***WH*** is an approximation of the input matrix ***I***.

In NMF [10], there is no guarantee that a physical phenomenon is directly reflected in the basis matrix. In order to analyze physical phenomena, a constraint on the basis function was introduced into BzNMF [13]. It decomposes the non-negative matrix into the inner products of non-negative basis vectors and non-negative coefficients, under the constraint that the decomposed basis vectors are represented by the Boltzmann distribution. Thus, BzNMF can directly estimate the model parameters of the target phenomena in the framework of NMF. It decomposes ***I*** into ***W*** and ***H*** as
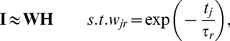
(5)where *w_jr_* is the *j*-th value of the *M*-dimensional basis vector ***w*** *_r_* and is expressed by the given model function in Eq. (1). BzNMF decomposes the input signal sequences by estimating the time constant in the *r*-th component (rank) *τ_r_* (>0) and the coefficient *h_ri_*.

The objective function in [13] minimized the generalized KL divergence (also referred to as I divergence), which was given by
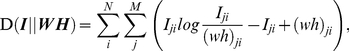
(6)where *I_ji_* and (*wh*)*_ji_* = ∑*_r_ w_jr_ h_ri_* are the *j*-th value of the *i*-th input vector and the *j*-th value of the *i*-th approximated vector, respectively. The objective function given by Eq. (6) represents the divergence between *I_ji_* and (*wh*)*_ji_*, and the objective function of the fitting method is usually represented using the LSE [14]. Thus, we propose an objective function for BzNMF to minimize LSE, as follows:
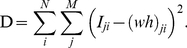
(7)In BzNMF, the time constants *τ_r_* and the non-negative coefficients ***h*** are optimized to estimate the approximations. From the objective function given in Eq. (7), the derivative with respect to *τ_r_* is obtained as follows:
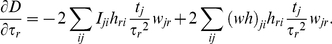
(8)We can derive the update rule for *τ_r_* using the step width parameter (acceleration coefficient) *η* of the gradient descent formula *τ_r_* ← *τ_r_* - *η*{∂*D*/∂*τ_r_*}. Similar to the original NMF, *η* (≥0) is as follows:
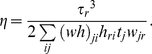
(9)The update rule for the coefficient *h_ri_* in the proposed BzNMF is the same as that for the original NMF. Thus, the update rule for the parameters in the proposed BzNMF is given as follows:
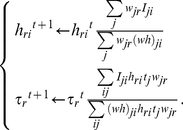
(10)The weighted component vector of the *r*-th component in the *i*-th signal sequence is represented as ***w*** *_r_h_ri_*. The proof of these update rules is the same as that for the original NMF [10].

### Rank Estimation using an Information Criterion

In spectroscopic analysis, the number of basis vectors (components), i.e., the rank, is usually determined manually based on chemical knowledge. However, automatic estimation of the rank is important because the rank affects the decomposition results. Rank estimation using *k*-fold cross validation (CV) [19] was presented in [13]. Cross validation is a popular model selection technique and evaluates models more directly than other theoretical methods, such as information criteria. However, the given parameter *k* depends on the user. If the input data is sufficiently given for statistical (theoretical) models, rank estimation using the information criterion is less computationally expensive than rank estimation using *k*-fold CV, because the information criterion can estimate the rank from a one-time-only validation. The computation times for AIC and *k*-fold CV are compared in the Results section. In the present paper, the rank is estimated using an information criterion.

Information criteria such as the AIC [15] and the minimum description length (MDL) [20], [21] are commonly used as model selection methods. In particular, the AIC is a simple information criterion and can be used to evaluate the goodness of the statistical distribution models. Therefore, among the numerous information criteria, we select the AIC for use in estimating the rank. The AIC minimizes the log likelihood and is expressed as follows:
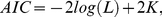
(11)where *L* and *K* are the likelihood and the degree of freedom of the model, respectively. In the rank estimation for the proposed BzNMF, *L* can be derived from the Gaussian distribution because the objective function is expressed using the LSE. In the proposed model (5), the degree of freedom is the rank *R*, which is estimated based on the AIC as
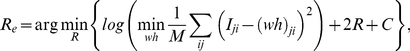
(12)where *R_e_* is the estimated rank, and *C* is a constant term.

When the objective function is based on the generalized KL divergence, the error distribution between the input signal sequence and the approximated signal sequence is assumed to be a Poisson distribution. In this case, rank estimation by the AIC is derived using Stirling's approximation,
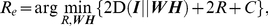
(13)where *D*(***I***∥***WH***) is the value of the objective function for each *R*.

If the number of input signal sequences is too small for rank estimation, the finite sample corrected AIC (AICc) [22] can effectively estimate the rank. The AICc is defined as
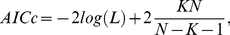
(14)where *N* is the number of signal sequences. When the objective function is based on the LSE, rank estimation by AICc is defined as follows:
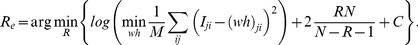
(15)Similarly, the AICc optimized in the generalized KL divergence is defined as follows:
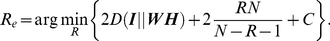
(16)In the following experiments, we use Eq. (12) in the proposed method.

## Results

### Comparative Evaluation of Factorization Methods

In this section, we use simulation data to compare decomposition methods that are optimized in the LSE or in the generalized KL divergence. The rank in the BzNMFs was estimated using the AIC. The input signal sequences were synthesized by the following equation,
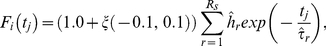
(17)where *ξ*(−0.1, 0.1) is random noise that ranges from −0.1 to 0.1. The simulation rank *R_s_*, the *r*-th time constant 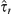, and the *r*-th existence ratio 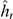 were given as *R_s_* = {2, 3, 4, 5}, 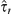 = 10*^r^*, and 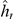 = 1/*R_s_*, respectively. In the simulation experiments, the ranks, time constants, and existence ratios estimated using the decomposition methods were evaluated using the mean values for three sets of simulation data. The input simulation matrix for each set was constructed from 50 vectors (signal sequences), which were equally sampled as 75-, 145-, 715-, 1,430-, and 7,150-dimensional (log sampling) vectors (1.002≤*t_j_*≤3,269,017.373). For rank estimation by the AIC, the AICc, and the *k*-fold CV, the range of given ranks was 1≤*R*≤20. Dataset S1 contains the source code of our proposed method (BzNMF, AIC and AICc optimized in the LSE) and the simulation data.

**Figure 1:**
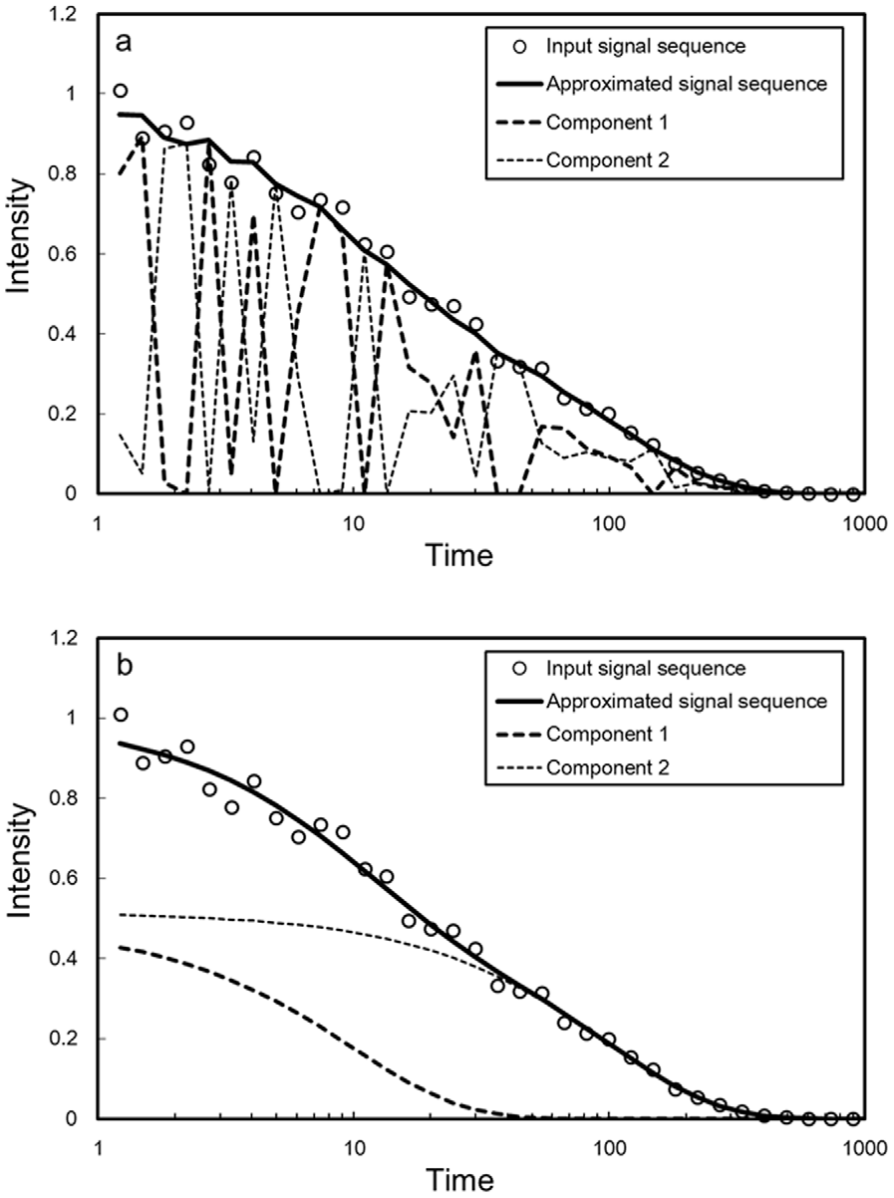
Decomposition results for the simulated signal sequence. Figure 1 shows examples of the decomposition results for one signal sequence. The input matrix is rank 2 and consists of 50 vectors (signal sequences). The signal sequence is represented by a 75-dimensional vector. The open circles, the solid line, and the broken lines show the input signal sequence, the approximated signal sequence, and the decomposed components, respectively. (a) shows the decomposition results obtained using the NMF optimized in the LSE. The rank of (a) is assumed to be 2. (b) shows the decomposition result obtained using BzNMF + AIC optimized in the LSE. The rank of (b) is estimated to be 2 using the AIC.

**Figure 2:**
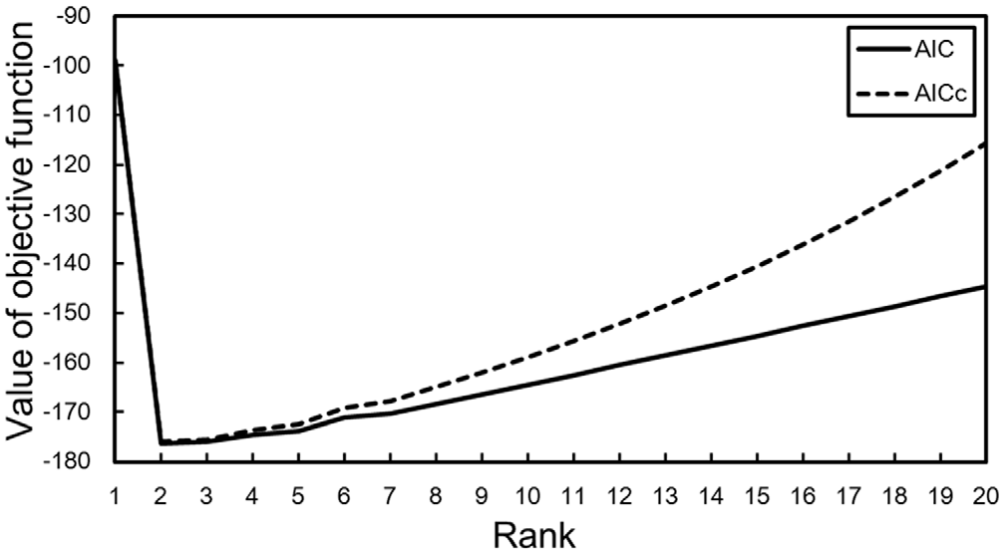
Rank estimation results by AIC and AICc. The input matrix setting is the same as Figure 1. The experimental results are obtained from one set of input matrices (50 signal sequences). The AIC and AICc are optimized in the LSE. The solid line and the broken line show the results obtained by the AIC and the AICc, respectively.

Figure 1 shows examples of the decomposition results obtained using the NMF [10] and BzNMF (10), which were optimized in the LSE. The open circles indicate the input signal sequences, and the solid lines indicate the approximated sequences. The broken lines indicate the weighted component sequences for each rank *r*. The rank of simulation data was given as 2. The rank was set to 2 in the decomposition using the NMF, which is not always suitable for factorizing composite physical signals, as shown in Fig. 1a, because model functions representing physical phenomenon are not introduced into the bases. On the other hand, BzNMF could factorize the modeled components as shown in Fig. 1b, and the proposed method using the AIC given by Eq. (12) was used to estimate the correct rank (2, in this case), as shown in Fig. 2.

Figure 2 shows the results of rank estimation using the AIC and the AICc, which were optimized in the LSE. The estimated rank was 2 using both the AIC and the AICc. Based on these results, the AIC can effectively estimate the correct rank when the number of input signal sequences is relatively small, i.e., approximately 50.

**Figure 3:**
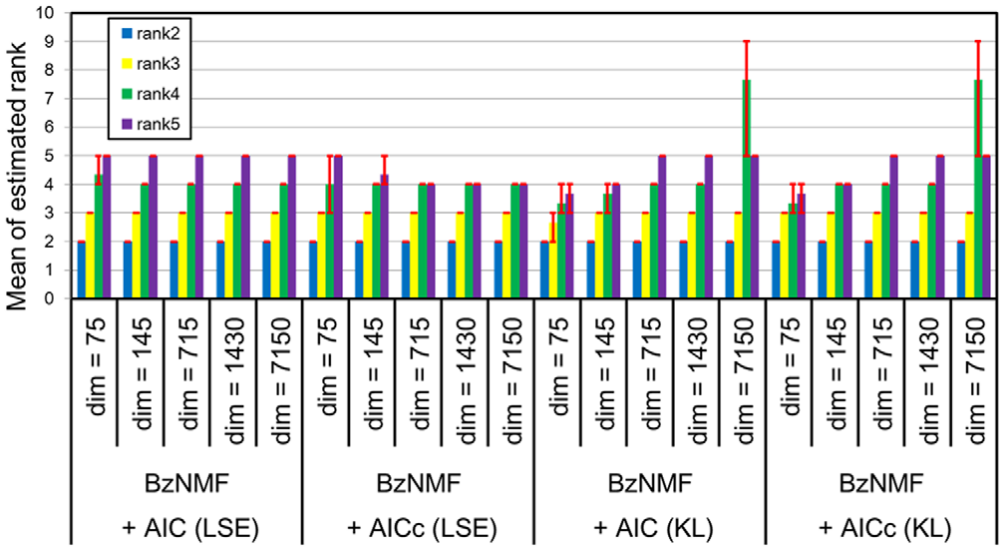
Rank estimation results for different ranks and sample dimensions. The simulation rank *R_s_* and the dimension of the signal sequence (*dim*) are set to *R_s_* = {2, 3, 4, 5} and *dim* = {75, 145, 715, 1,430, 7,150}, respectively. The input matrix is constructed from 50 signal sequences in a set. The ranks are estimated by three sets of input matrices. The blue, yellow, green, and purple bars show the mean of estimated ranks in *R_s_* = 2, *R_s_* = 3, *R_s_* = 4, and *R_s_* = 5, respectively. The red error bars show the maximum and minimum estimated ranks.

In order to confirm the estimation accuracy, we evaluated the ranks, existence ratios, and time constants estimated using three sets of input matrices, which were constructed from 50 simulated signal sequences in one set. Figure 3 shows the results of rank estimation obtained using the automatic decomposition methods for each *R_s_* and the dimension of the signal sequence (*dim*). The automatic decomposition methods were BzNMF (10) and the AIC (12) optimized in the LSE (BzNMF + AIC (LSE)), BzNMF (10) and the AICc (15) optimized in the LSE (BzNMF + AICc (LSE)), BzNMF [13] and the AIC (13) optimized in the generalized KL divergence (BzNMF + AIC (KL)), and BzNMF [13] and the AICc (16) optimized in the generalized KL divergence (BzNMF + AICc (KL)).

As indicated in Fig. 3, BzNMF + AIC (LSE) is the most stable method of rank estimation, because this method estimates the correct ranks, excluding one of the three trials in the case of the (*R_s_*, *dim*) = (4, 75) simulation data set. Unfortunately, BzNMF + AICc (LSE) could not estimate the correct ranks when *R_s_* = 5 (145≤*dim*≤7,150) and (*R_s_*, *dim*) = (4, 75). The false results for the AICc may arise from the effect of the finite sample correction term. BzNMF + AIC (KL) and BzNMF + AICc (KL) show the correct results when *dim*≥715, excluding *R_s_* = 4. In the case of (*R_s_*, *dim*) = (4, 7,150), the automatic decomposition methods optimized in the generalized KL divergence could not estimate the correct rank. These results suggest that the proposed method (BzNMF + AIC (LSE)) is better than the other methods for automatic rank estimation.

For evaluating the estimated parameters by BzNMF, the estimated *τ_r_* and the coefficients (existence ratios) of the components *h_r_* are listed in Table 1. These are the results for (*R_s_*, *dim*) = (3, 145) shown in Fig. 3. When the input matrices in Table 1 were decomposed, the methods shown in Fig. 3 could correctly estimate the multiple ranks in the lower-dimensionality signal sequences. The parameters estimated by BzNMF + AICc were identical to those obtained by BzNMF + AIC, which were optimized by the same method. The decomposition results for the proposed method (BzNMF + AIC (LSE)) were similar to those for BzNMF + AIC (KL). The time constants *τ_r_*, in particular, *τ*_1_ and *τ_2_*, of BzNMF + AIC (KL) were slightly smaller (faster) than those of the correct values. On the other hand, the proposed method estimates the parameters with sufficient accuracy, indicating that the estimated parameters exist within the error range due to random noise shown in Table 1.

**Table 1:** Estimated parameters.

	Simulation parameters	BzNMF + AIC (LSE)	BzNMF + AIC (KL)
*τ*_1_ [st. dev.]	10	10.3700 [±0.3355]	8.9300 [±1.8650]
*h*_1_ [st. dev.]	0.33	0.3312 [±0.0176]	0.3331 [±0.0029]
*τ*_2_ [st. dev.]	100	99.1267 [±3.0792]	87.9633 [±2.2115]
*h*_2_ [st. dev.]	0.33	0.3335[±0.0167]	0.3364[±0.0017]
*τ*_3_ [st. dev.]	1000	998.5633 [±4.5576]	998.3933 [±21.5003]
*h*_3_ [st. dev.]	0.33	0.3352 [±0.0078]	0.3305 [±0.0034]

The estimated time constant (*τ_r_*) and existence ratio (*h_r_*) are shown as the mean of the results shown in Fig. 3 (*R_s_*, *dim*) = (3, 145). The results obtained by BzNMF + AIC and BzNMF + AICc are the same when the optimization criterion is the same.

**Figure 4:**
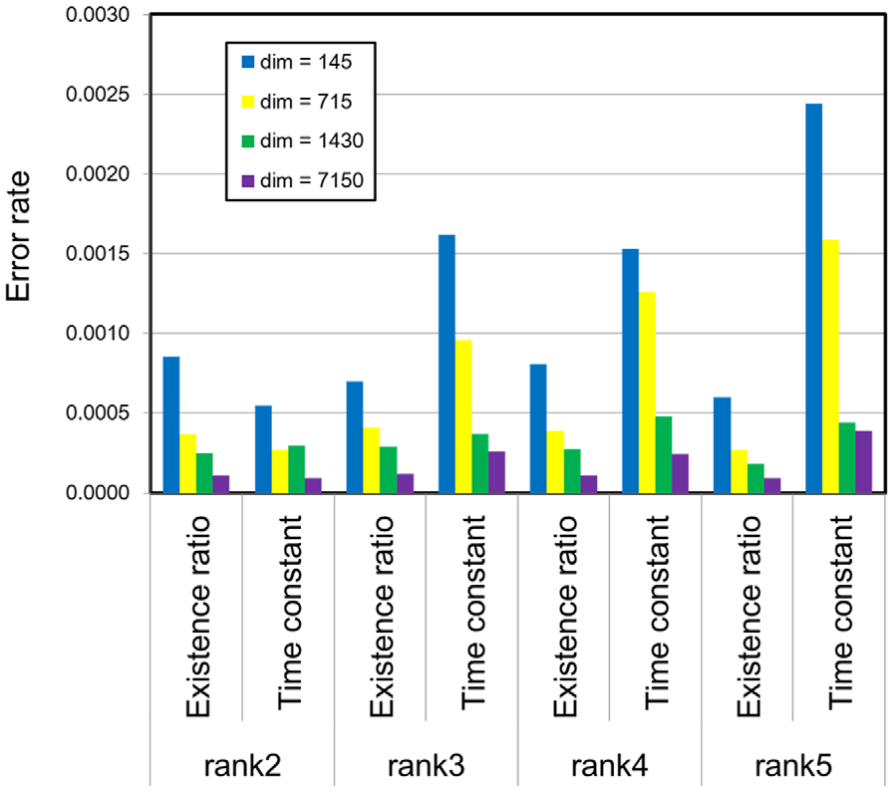
Error rates of parameters estimated by BzNMF + AIC optimized in the LSE. The simulation rank *R_s_* and the dimension of signal sequence (*dim*) are set to *R_s_* = {2, 3, 4, 5} and *dim* = {145, 715, 1,430, 7,150}, respectively. The input matrix is constructed from 50 signal sequences in a set. The parameters (existence rate and time constant) are estimated from the three sets of input matrices. The error rates of the parameters are calculated from 50×3 signal sequences (error rate of existence rate) and three sets of input matrices (error rate of the time constant). The blue, yellow, green, and purple bars show the averaged error rates for *dim* = 145, *dim* = 715, *dim* = 1,430, and *dim* = 7,150, respectively.

In order to evaluate the influence of the input signal dimensionality on the decomposition parameters, the error rates of the estimated parameters by BzNMF + AIC (LSE) are shown in Fig. 4. The simulation parameters in Fig. 4 are the same as those in Fig. 3, where the ranks were correctly estimated using BzNMF + AIC (LSE). The error rates were calculated as
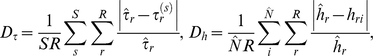
where *D_τ_* and *D_h_* are the error rates for the time constant and the existence ratio, respectively, *S* is the number of input matrix sets, 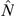 = *N* × *S* is the number of total input signal sequences, 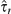, 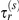, 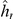, and 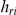 represent the *r*-th given simulation time constant, the *r*-th estimated time constant in the *s*-th input matrix, the *r*-th given simulation existence ratio, and the *r*-th estimated existence ratio in the *i*-th signal sequence, respectively. These results reveal that the parameter estimation accuracies increased in proportion to the number of dimensions.

Thus, BzNMF + AIC (LSE) can correctly decompose composite physical signals expressed by the Boltzmann distribution law and can automatically estimate the correct rank when the dimension of the signal sequence is sufficiently large (*dim*≥75).

Finally, we compared the computation time (CPU time) and the rank estimation results for BzNMF + AIC (LSE) and BzNMF (10) and *k*-fold CV optimized in the LSE (BzNMF + *k* CV (LSE)), as shown in Table 2. The parameter *k* in CV was set to 3. The CPU times and the estimated ranks were evaluated by the mean values of the three sets of input matrices, similar to Fig. 3, for *dim* = 145. The CPU times were measured using an Intel Core i7 980×(3.33 GHz) processor. Based on the results, BzNMF + AIC (LSE) was approximately twice as fast as BzNMF + *k* CV (LSE) and could estimate the correct rank even when BzNMF + *k*CV (LSE) failed, as shown in Table 2 (simulation rank: 3, 4, 5). Rank estimation by *k*-fold CV becomes increasingly difficult because the number of decomposed signal sequences in the matrix decreases with *k*. When *k* is set to be greater than 3, the rank estimation accuracy by *k*-fold CV may be improved, because the number of decomposed signal sequences increases. However, the CPU times for CV are likely to increase with *k*. Thus, rank estimation using the AIC is better than that using *k*-fold CV.

**Table 2:** Comparison of computation times and estimated ranks.

	BzNMF + AIC (LSE)		BzNMF + *k*CV (LSE)	
Simulation rank	CPU time [st. dev.] (sec.)	Mean of estimated rank	CPU time [st. dev.] (sec.)	Mean of estimated rank
rank2	**397.00 [±12.37]**	**2**	854.90 [±38.84]	**2**
rank3	**419.43 [±18.72]**	**3**	867.96 [±44.42]	9.33
rank4	**422.25 [±67.05]**	**4**	832.85 [±23.87]	9.33
rank5	**380.38 [±17.65]**	**5**	855.02 [±28.34]	9.67

The computation times (CPU times) and the estimated ranks are evaluated using three sets of input matrices, similar to the case for Fig. 3 (*dim* = 145). Parameter *k* in CV is set to 3.

### Application to Real Data

We next applied the proposed method to real signal sequences, which were measured based on chemical particle dynamics in an aqueous solution (Rh6G) and protein dynamics in living cells (EGFP). These signals were measured using FCS [3] and were fitted using an FCS model function [23]. The model function (Eq. (23) in [23]) was constructed using terms for free diffusion of particles and a chemical reaction such as unimolecular isomerization or energy transfer from a higher excited state. The free diffusion term was determined from the time-series deviation of the particle concentration, which was obtained by taking the Fourier transform, and the chemical reaction term was expressed using the Boltzmann distribution, as shown in Eq. (1). We compared the results obtained by BzNMF + AIC (LSE) (proposed method) and those obtained by the fitting method [23]. The input signal sequences were normalized by linear regression when the signal sequences were decomposed using the proposed method.

The Rh6G signal is assumed to consist of two components, based on chemical knowledge. The main component is derived from free diffusion in the aqueous solution, and the other component represents energy transfer from a higher excited state. In the present study, the concentration of the Rh6G aqueous solution was 10^−7^ mol/ℓ. The input matrix consisted of 54 signal sequences, each being represented by a 92-dimensional vector (1.6≤*t_j_*≤4505.6). Similar to the case for the Rh6G signal, based on chemical knowledge, the EGFP signal is theoretically assumed to consist of two components. However, based on biological knowledge concerning living cells, the EGFP signal is conjectured to consist of three or more components, because free diffusion of EGFP can be self-inhibited and/or inhibited by intracellular structures [24]. The concentrations of EGFP in living cells are uncontrollable and exhibit a wide range of variation. The input matrix consisted of 44 signal sequences, each represented by a 101-dimensional vector (6.0≤*t_j_*≤36044.8). In chemical and biological fields, when signal sequences are decomposed using the fitting method, the ranks are usually determined as the minimum values from the heuristics [1]–[4]. In EGFP in particular, the estimated time constant in the main component was approximately the same when the given ranks of the fitting method were changed [3]. Therefore, the ranks of the fitting method were determined to be 2 for decomposition of signals from Rh6G and EGFP. For rank estimation using the proposed method, the range of given ranks *R* was the same as that in the previous section, 1≤*R*≤20.

**Figure 5:**
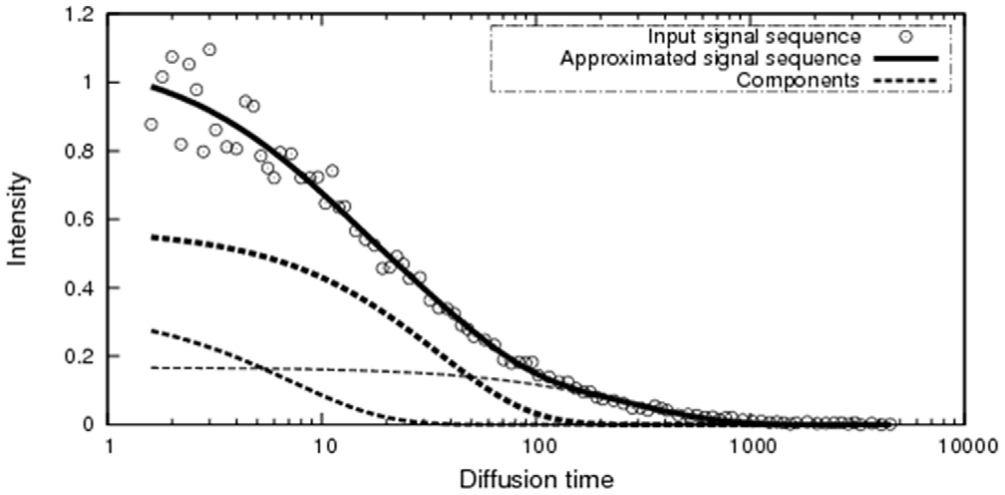
Decomposition results for the Rh6G signal sequence. Figure 5 shows an example of the decomposition results for one signal sequence. The input matrix is the Rh6G measurement data in aqueous solution and consists of 54 signal sequences. The signal sequence is represented by a 92-dimensional vector. The rank was estimated to be 3 using the AIC. The open circles, the solid line, and the broken lines show the input signal sequence, the approximated signal sequence, and the decomposed components, respectively.

Figure 5 shows an example of decomposition results for Rh6G obtained using the proposed method. The open circles, solid lines, and broken lines are as described in Fig. 1. The rank was estimated to be 3 using the AIC. Note that the proposed method could clearly decompose the basis vectors (components) and the approximated vector was a reasonable fit to the noisy input data, as shown in Fig. 5.

Decomposed parameters such as the time constants and the existence ratios for Rh6G are shown in Table 3. The parameters obtained using the fitting method [23] were fitted assuming two components that represent the energy dynamics in the high excitation state (1^st^ component) and the free diffusion of particles (2^nd^ component). The results of rank estimation obtained by the AICc were the same as those obtained by the AIC. The estimated time constant and existence ratio of the 1^st^ component obtained by the proposed method are the same as those obtained by the fitting method. The 2^nd^ component is the primary component, and the time constant of the proposed method exists in the error margin of the 2^nd^ component of the fitting method. The 3^rd^ component of the proposed method may correspond to the slow diffusion of particles in the aqueous solution, because the time constant and the existence ratio were sufficiently slow and low, respectively, compared to the 2^nd^ component. These results indicate that the estimated rank and parameters are reasonable values based on chemical knowledge.

**Table 3:** Estimated parameters for the Rh6G input matrix.

Fitting Method [23] (Given rank is 2)		Our proposed method (Estimated rank is 3)	
*τ*_r_	Existence ratio	*τ*_r_	Existence ratio
**7.23±13.95**	**0.328±0.285**	**7.24**	**0.292±0.046**
**24.89±11.49**	1.0	**34.84**	0.547±0.047
		279.80	0.161±0.012

The results are evaluated using the Rh6G input matrix, which consists of 54 signal sequences, each of which is represented by a 92-dimensional vector. The rank of the fitting method [23] is set to 2 based on chemical knowledge, and the rank of the proposed method is automatically estimated using the AIC (12). In the fitting method [23], the time constants and the existence ratios are the mean values of 54 signal sequences.

The obtained parameters for EGFP are listed in Table 4. The parameters for the fitting method [23] were obtained by assuming two components, for the same reasons as in the case of Rh6G. The rank obtained by the proposed method was estimated to be 4 using the AIC. The rank estimation results for the AICc were the same as those for the AIC. The parameters of the 1^st^ component estimated using the proposed method are the same as those obtained using the fitting method. However, the 2^nd^ time constant obtained by the proposed method is faster than that obtained by the fitting method. The reasons for the faster time constant in the 2^nd^ component may be as follows. The proposed method assumes non-negative signal sequences that are represented as sums of Boltzmann distributions. The fitting method [23] is derived from the spectroscopic model, which is not the same as the Boltzmann distribution (2). Thus, the parameters estimated by the proposed method are not necessarily the same as those estimated by the fitting method. Moreover, the free diffusion of EGFP may not follow the ideal Boltzmann distribution law, because EGFP has a tendency to aggregate depending on the *pH* of the aqueous solution and the concentration of EGFP [24]. In biological experiments, the concentration of EGFP is very difficult to control in living cells. The 3^rd^ and 4^th^ components obtained using the proposed method may also represent inhibited diffusion of proteins resulting from self-binding and/or interactions between EGFP and intracellular structures.

**Table 4:** Estimated parameters of the EGFP input matrix.

Fitting method [23] (Given rank is 2)		Our proposed method (Estimated rank is 4)	
*τ*_r_	Existence ratio	*τ*_r_	Existence ratio
**32.70±26.73**	**0.169±0.075**	**30.38**	**0.149±0.038**
243.57±53.44	1.0	165.06	0.469±0.034
		846.44	0.321±0.042
		11429.07	0.061±0.016

The results are evaluated using the EGFP input matrix, which consists of 44 signal sequences. The signal sequence is represented by a 101-dimensional vector. The rank of the fitting method [23] is set to 2 based on previous biological knowledge, and the rank of the proposed method is estimated automatically using the AIC (12). In the fitting method [23], the time constants and the existence ratios are the mean values of 44 signal sequences.

The proposed method (BzNMF + AIC) and the fitting method [23] both estimate reasonable parameters for the real data by referring to heuristics. In particular, the proposed method statistically decomposes the signal sequence into physical components, because the parameters of physical model functions and the number of components (rank) are automatically estimated from numerous signal sequences. Thus, the proposed method is widely applicable to data analysis in the case of unknown rank.

## Discussion

We proposed an automatic decomposition method for analyzing composite physical signals. This novel method uses non-negative factorization and includes a model function that follows the Boltzmann distribution law. Furthermore, the proposed method can automatically estimate the rank using the AIC.

In the analysis accuracy verification using simulation data, the proposed method provided better factorization results than the original NMF [9], [10] and better results compared with BzNMF, in which the objective function was based on the generalized KL divergence. In addition, the proposed method automatically estimates the rank using the AIC, which has a lower computational cost than the rank estimation method for *k*-fold CV.

In the analysis of real data, the most important thing is that the automatically estimated parameters are reasonable in terms of heuristics such as the results of biological and/or chemical experiments. The proposed method automatically and statistically decides the rank and the parameters of the model functions. The rank in the fitting method [23] is set manually as a minimal value from the heuristics in order to simplify and explain the meaning of the decomposed components. However, manual rank decision is difficult for unknown samples and does not necessarily guarantee the true rank. In the case of unknown samples, the rank should be decided based on the statistics of the input samples without a manual rank decision so as to ensure the repeatability of the analytical results.

As shown by the experimental results for the real data, the proposed method achieves acceptable results for the Rh6G samples, as compared with the fitting method [23], and automatically estimates reasonable parameters based on chemical and biological knowledge, as in the case of the EGFP samples. Thus, the proposed method is useful for automatic analysis of composite physical signals that follow the Boltzmann distribution law.

## Supporting Information

Dataset S1 **CodeAndSample.zip.** CodeAndSample.zip contains the source code of our proposed method and the simulation data in this article.
(ZIP)
